# The Australian 2019/2020 Black Summer Bushfires: Analysis of the Pathology, Treatment Strategies and Decision Making About Burnt Livestock

**DOI:** 10.3389/fvets.2022.790556

**Published:** 2022-02-15

**Authors:** Brendan D. Cowled, Melanie Bannister-Tyrrell, Mark Doyle, Henry Clutterbuck, Jeff Cave, Alison Hillman, Karren Plain, Caitlin Pfeiffer, Michael Laurence, Michael P. Ward

**Affiliations:** ^1^Ausvet Pty Ltd., Bruce, ACT, Australia; ^2^Faculty of Science, Sydney School of Veterinary Science, University of Sydney, Camden, NSW, Australia; ^3^South East Local Land Services, Goulburn, NSW, Australia; ^4^Biosecurity and Agriculture Services, Agriculture Victoria, Wodonga, VIC, Australia; ^5^Faculty of Veterinary and Agricultural Science, Melbourne Veterinary School, University of Melbourne, Parkville, VIC, Australia; ^6^Meat and Livestock Australia, North Sydney, NSW, Australia

**Keywords:** Australia, bushfire, wildfire, livestock, injury, risk factors, euthanasia, decision making

## Abstract

In 2019/2020, Australia experienced a severe bushfire event, with many tens of thousands of livestock killed or euthanized. Little systematic research has occurred to understand livestock bushfire injuries, risk factors for injury, or how to make decisions about management of bushfire-injured livestock. Addressing this research gap is important as there is an increasing bushfire incidence globally. This paper presents qualitative research findings about bushfire-injured and killed livestock in the south-east of Australia after the 2019/2020 Australian bushfires. We describe observed pathology, treatments used, and risk factors for injury, then use thematic analysis to understand decision making about managing fire-injured livestock. Livestock injured by the fires showed pathology predominantly associated with the common integument (feet, hooves and skin) and signs of acute respiratory damage. It could take several days for the full extent of burns to become apparent, leaving prognostic doubt. Treatment strategies included immediate euthanasia, salvage slaughter, retention for later culling, treatment and recovery on farm, hospitalization and intensive treatment, or no intervention. Risk factors reported for livestock injury included lack of warnings about an impending fire, the type and amount of vegetation around livestock and the weather conditions on the day the fire reached livestock. Moving stock to an area with little vegetation before fire arrived was seen as protective. Decision making regarding injured livestock appeared influenced by three main themes: (1) observations on the severity of pathology, clinical signs and level of prognostic doubt, (2) pre-existing beliefs about animal welfare (responsibility to minimize unnecessary suffering) and (3) assumptions about the future. The management of livestock was largely appropriate due to the rapid provision of veterinary expertise. However, it is likely that some injured livestock were euthanized due to conservative veterinary advice driven by a lack of opportunity to re-assess stock, with impacts on farmers. In future, resourcing regular revisits of injured livestock to manage risks of gradual progression of burn pathology may facilitate more accurate prognostic assessment, provided injured animals can receive appropriate pain relief. In addition, a more comprehensive burns classification system linked to prognosis that can be rapidly applied in the field may assist assessments.

## Introduction

Australia had its hottest and driest year on record in 2019 and endured a series of heatwaves over much of Australia in December 2019 (1). In the lead up to this, much of southeast Australia had suffered a protracted drought from 2017 with rainfall values in New South Wales (NSW) and southern Queensland near or below previous record low values (1). The accumulated Forest Fire Danger Index in spring 2019 was significantly higher than in any other spring on record (1). Then in the spring and summer, Australia experienced a severe bushfire event. During this bushfire event more than 19 million hectares of land burnt, more than 3,000 homes were destroyed and 33 people died (1, 2). It was estimated that the fires and exposure to particulate matter led to several hundred excess human deaths and thousands of hospitalizations (3).

It was estimated in the media that more than 56,000 livestock were killed by fire or euthanized in NSW, Victoria and South Australia (4). However, the true impact on livestock and livestock production is unknown. Despite this, the livestock population at risk in these areas is relatively extensive indicating a small proportion of stock was lost. For example, livestock population data (5, 6) in bushfire-affected regions of NSW and Victoria indicate that there were 3.6 million cattle and 21 million sheep in bushfire-affected regions, although many would not have been close to fire within those regions because of the coarse scale of the population data (BC, unpublished data). The local impact on some individual farmers was very high. For example, in a recent case control study, some farms suffered an impact of up to $2 million (AUD) and deaths of all livestock on a farm (BC, unpublished data).

Bushfires (wildfires) are increasing in frequency globally, especially as a result of longer fire seasons in temperate or boreal regions (7, 8). Little research has been conducted on the impacts of bushfires on livestock in any part of the world. For example, a systematic literature review by co-authors (BC, AH and CP) revealed barely a dozen publications, mostly case studies in Australia (9–22). More specific published research on pathology, injuries and risk factors for burns due to bushfire are even more limited. This paucity of literature limits understanding and the ability to manage bushfire affected stock in an optimal way. This is especially concerning given the increasing frequency and severity of such events.

It can be impossible or difficult to collect field data during bushfire emergencies. Under such circumstances it is difficult to collect data as veterinary and research resources are scarce or difficult to deploy. Qualitative research methods seek to uncover a diversity of views and meanings that people bring to an issue under investigation (23). Such approaches can provide the veterinary profession with insights into topics that are hard to reach with more widely used quantitative research methods (23), such as observational epidemiological studies.

The objectives of this study were:

1. To describe the pathology, treatment strategies, treatments practically used, and risk factors for injury reported by assessing veterinarians
2. To analyze the decision making by veterinarians assessing and advising on bushfire affected livestock, especially how they decided whether to treat or euthanize bushfire-injured livestock.

## Materials and Methods

A qualitative study was implemented to gather data to understand the perspectives and decision-making of professional veterinarians when assessing and responding to bushfire-affected livestock. The methodology reported here is structured to comply with the Consolidated Criteria for Reporting Qualitative Research (COREQ) (24).

### Research Team and Reflexivity

Three authors (BC, MW and MB-T) developed the semi-structured interview guide independently of other authors (see Supplementary Material). The interviews were conducted by the lead author (BC). The analyses were first conducted by BC with subsequent assistance and commentary from all co-authors. BC is a male veterinary epidemiologist (PhD, FANZCVS) and beef producer who was from a bushfire affected farm. MB-T is a female medical epidemiologist (PhD) who has extensive experience in qualitative and mixed methods epidemiology and strategically assisted in the project to ensure methodologies were well-implemented. MW is a male veterinary epidemiologist (Ph.D., FANZCVS) and has used qualitative methods in veterinary epidemiology for several years.

The interviewer (BC) established a new professional relationship with most of the veterinarians interviewed for the purposes of the study. However, BC had worked with three of the veterinarians during other projects in the past. Participants were aware of the interviewer's qualification and background through advanced notice and information about the study, and the interviewees were advised about BC's credentials and experience when the interview occurred.

### Study Design

#### Informant Veterinarian Selection

**Figure 1:**
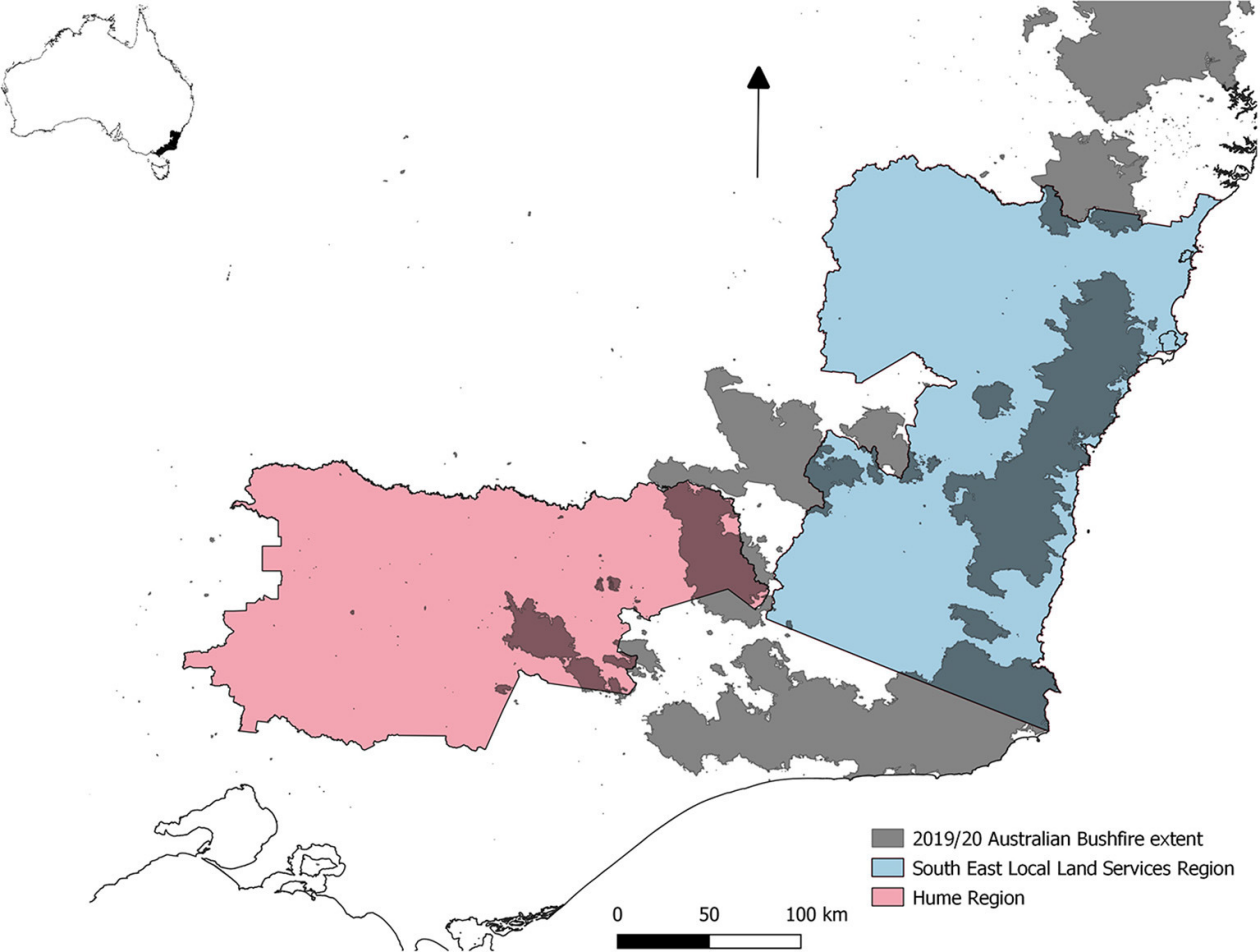
A map displaying the two regions of Australia where the study occurred with bushfire extent overlaid. The location of the study site within the Australian continent is inlaid.

This research purposively selected a geographic area from which to sample key informants (veterinarians). This area was in the southeast of NSW and in northern Victoria, and was the main bushfire-affected region in south east Australia in December 2019 and January 2020. This included the following districts: Bega, Bombala, Braidwood, Goulburn, Milton/Kangaroo Valley, and Riverina in the Southeast Local Land Services region of NSW, and Upper Murray district in the Hume region of Victoria. These regions are shown in Figure 1 with overlying bushfire extent. The government district veterinarian from each district within the regions that responded to fire in the 2019/20 bushfire season were included in the sampling frame for this research. In addition, a privately employed veterinarian was also interviewed, on recommendation from a local district veterinarian due to their extensive involvement during the fire response. Thus, eight veterinarians were contacted by email and telephone calls and all participated and were interviewed.

Veterinarians all had at least 3 years of rural clinical experience and had attended at least one bushfire response. Four veterinarians had attended fires during two or more bushfire seasons, with one attending fires for 20 seasons, including as a professional fire fighter. Four veterinarians had only attended fires in one season, the 2019/2020 season. All veterinarians individually visited at least 5 and up to 100 (mean 49 farms, 95% CI: 20–78) bushfire-affected farms in the 2019/2020 season.

Veterinarians were encouraged to speak about all bushfires they had attended as veterinarians to assess and treat livestock, not just the 2019/2020 fires.

#### Data Collection

The same interview guide was used throughout all eight interviews. Main sections of the interview were: background information about the respondent; descriptions of pathology seen; treatment of burnt livestock; protective factors on-farm that may have prevented injuries; and farm recovery. See Supplementary Material where the interview guide is detailed. The questions in the interview guide were asked as open-ended questions of the informant veterinarians. Each interview lasted 30–60 min.

The interviews occurred in the second half of calendar year 2020, ~9–12 months after the end of the bushfires in the 2019/2020 season. Three interviews occurred face to face. COVID-19 precautions resulted in the remaining five interviews being conducted by online video-conferencing (Zoom: https://zoom.us/). The face-to-face interviews occurred in the informant veterinarians work setting (office). Interviews were one-on-one with just the interviewer (BC) and informant veterinarian present.

Four of the informant veterinarians were men and four were women. Their median age was 41 years (range: 28–55).

Where clarifications were required after interviews, veterinarians were contacted *via* email or phone calls. This occurred on three occasions.

Interview audio recordings were transcribed initially using Amazon Web Services (https://aws.amazon.com/) and the Amazon Transcribe tool, which uses machine learning. Automatically transcribed word documents were manually checked and corrected by iteratively listening to and correcting transcripts. These transcripts were viewed only by the research team and were not returned to informant veterinarians. The manuscript was returned to three key informants for comments.

#### Descriptive and Data Analysis

An initial descriptive analysis focused on identifying and describing the pathology, treatment strategies, treatments used and risk factors identified by the informants. An inductive, semantic thematic analysis [following the approach of Braun and Clarke (25)] to explore the decision making processes veterinarians used to decide how to respond to bushfire-injured livestock.

The interview records were imported into NVIVO, release 1.3 (https://www.qsrinternational.com/nvivo-qualitative-data-analysis-software/home). During coding, the interview material was read and re-read, and each idea related to the research question was identified and marked or coded in NVIVO. Themes were derived from the code groupings. Subthemes were organized within themes. Several iterations occurred before final themes and subthemes were identified. The sub-themes described exhaustively the ideas identified in the interview materials with the exception of farm recovery. This topic was arbitrarily excluded from analysis for brevity and because the content did not align or enhance the emergent themes in the rest of the interview material.

#### Ethics

This research was approved by The University of Melbourne's Human Research Ethics Committee (ethics ID 2057893.1). Key researchers (BC, CP) underwent trauma informed care training. A plain language statement was provided to informants that described the research project, approach, what the research was about, the informant's role, withdrawal, and possible benefits of the research. A verbal consent form was developed and administered to informants.

## Results

These results are divided into two parts. The first part is the descriptive results, namely the simple description of what veterinarians told us about the key areas of interest (e.g., observed pathology). The second part presents analytical findings about how veterinarians decided whether to euthanize or treat bushfire-injured livestock.

### Descriptive Analysis

The interview material provided several topic areas when conducting descriptive analyses. This included:

- External gross pathology and clinical signs
- Possible treatment outcomes
- Risk and protective factors for bushfire exposed livestock injury.

These topics are described in the following sections.

#### External Gross Pathology and Clinical Signs

This includes information that describes the wide spectrum of gross pathology in bushfire affected sheep and cattle observed by attending veterinarians. A key finding was the limited ability for informant veterinarians to examine anything other than gross external pathology, as post mortem examinations and other investigations were not conducted due to the nature of the emergency. There was a wide spectrum of severity of injury reported.

##### Context in Terms of Prevalence of Dead and Euthanized Livestock

Data describing the number of cattle that either died due to burns or were euthanized in the fires in 2019/2020 in their district were provided by one veterinarian only and are reported here. Similar data were not accessed from other districts. In the upper Murray district there are approximately 55 000 cattle. 3 580 cattle (7%) were euthanized or died due to bushfire burns. Nearly all the district farming land burned during the fire, and there were extensive areas of bush. This means that most cattle had relatively close contact with fire, sometimes very severe fire. There were 2 053 sheep killed in the upper Murray district, but the underlying sheep population was not able to be accurately estimated.

Of the 3,580 cattle killed during bushfires, only 373 (12%) were euthanized by Agriculture Victoria (the state agricultural department) after veterinary assessment and 88% died due to burns or were euthanized by farmers. Of 2 052 sheep killed during the bushfires, 473 (23%) were euthanized by Agriculture Victoria after veterinary assessment and 77% died due to burns with no intervention or were euthanized by farmers.

##### Veterinarians Can Only Report on External Pathology

Attending veterinarians were operating in a disaster area and noted they were working with various practical and legal impediments including:

- Bushfires were still active leading to dangerous conditions that limited farm access
- Navigation was difficult as often veterinarians were operating in unfamiliar areas, road signs and recognisable features had burnt, or databases of farm locations were not available due to telecommunications failures.
- States of emergency and other legal controls were declared and veterinarians could not always access farms without escorts or permission, and veterinary access was not always treated as high priority. One veterinarian was appreciative of being kept safe, but explained that it delayed their response:
  “*So there was an incident response at the council, and the incident controller was police*.
  *Yeah he came out here and said you can't go out. Then they had a couple of priority calls and we were escorted out. But it just took a while for the wheels to start turning. And then it was only at the point where things were really safe. You don't mind them trying to keep me safe, but a lot of decisions had already been made in that in that time frame, without advice.”* [Interviewed Veterinarian (IV1)]
- There was a high workload with hundreds of farms to visit in a district and only a limited animal health workforce.

Together, these factors resulted in veterinarians having a limited time available to attend a bushfire affected farm, although the veterinarians visited up to dozens or hundreds of farms each. Thus, veterinarians were only able to perform an external examination of bushfire affected livestock and no information is available from veterinarians on the possible internal gross pathology associated with bushfire affected stock (e.g., pathology affecting the lungs and pleura). In addition, several informant veterinarians reported that a small proportion of stock on farms were examined from a distance as stock handling facilities were burnt. Thus, there is only a detailed understanding of the external gross pathology associated with bushfires from this study.

For example, one veterinarian stated “*there are always a number of animals to assess and you are always under that bit of time pressure, whether it's on the property that you're on, that you're having to get through the animals that are damaged or whether it's because you've got to get onto another property. So, no, I've never sort of stopped to see things further.”* [IV2]

##### Anatomical Area Affected by Fire

Areas observed with signs of pathology included the common integument such as hooves and skin and associated structures (such as udder and teats), eyes and the respiratory system.

The most common areas affected by fire were the hooves, from mild burns of the coronary band (periople) or heel bulb to complete sloughing of the hoof. The periople is the narrow strip along the coronary dermis border that is at the junction between skin and wall of hoof above the hoof (26). However, all veterinarians interviewed that spoke of this area referred to it as the coronary band, similarly to horses. The authors thus generally refer to this area as the coronary band within this study.

Skin burns were also relatively common, especially burnt teats. Teats were reported to easily injured structures with good likelihood of healing, but with occlusions to milk flow frequently limiting the affected cow or ewe's later productive value (i.e., offspring will later starve when born as milk flow occluded). Large sections of burnt skin were rarer. Cases with large areas of burnt skin were associated with stock grazing in heavily vegetated areas, which was uncommon. However, skin burns varied from a small proportion of the body to 100% of the skin burnt and from superficial to full thickness burns.

Areas of skin that were close to the ground or not protected by wool or hair were more susceptible to burns. This included the axillary and inguinal areas, scrotum, prepuce, udders, vulva and around the legs and feet.

Clinical signs of respiratory injury were reported to be relatively frequent and were generally acute in nature. It is uncertain what gross respiratory pathology was occurring as no livestock were examined post-mortem. However, clinical signs of dyspnoea, exercise intolerance, increased respiratory rates, open mouthed breathing, frothy nasal discharge and death indicates that the pathology was sometimes likely very severe. In addition, there were many cases of nasal discharge and dyspnoea without severe signs or death.

Eye injuries were rarer and included subsequent pink eye or corneal burns. Some corneal burns were so severe that the animal was blinded with scar tissue, evident in surviving cattle sometime after the burns occurred.

##### Severity of Gross Pathology and Clinical Signs

There was a very wide spectrum of external gross pathology observed by veterinarians. This has been categorized into mild, moderate and severe pathology in this study based on their likely prognosis, although there is a continuous spectrum. Others have classified similarly (18). A mild classification indicated that livestock were injured in a minor way and could be retained on the farm for later breeding or managed culling when suitable. A moderate classification indicated that livestock were more severely injured, with cattle requiring salvage slaughter or adequate treatment and nursing on the farm. A severe classification indicated that if livestock were not already dead they would require immediate euthanasia.

These definitions do not include categorization based on the severity of animal welfare impacts. It is plausible and accepted that bushfire injuries in livestock affect animal welfare, sometimes very severely (11, 17, 18). However, there is insufficient previous research to understand the relative degree of suffering associated with different bushfire injuries. Injuries classified as moderate or severe clearly have substantive welfare impacts, and some negative welfare effect is likely also present for mild injuries.

- Mild

Gross pathology in mild cases included singeing of hair (cattle) and wool (sheep) or small superficial burns to exposed skin (e.g., vulva, inguinal and axillary areas and udders). In addition, some foot burns were evident that led to mild laminitis and lifting of the heel bulb, and clinical signs associated with weightbearing lameness.

Some subtle respiratory signs could be evident (e.g., serous or mucous nasal discharge) indicative of respiratory pathology.

Another clinical sign observed in mild cases was that some livestock were quiet and depressed and not as active as usual, or were generally stiff when moving. When examined, there was no obvious gross pathology (e.g., burnt feet). One veterinarian ascribed these signs to a possible generalized myopathy due to running from fire.

- Moderate

Moderate gross pathology included burns to various parts of the common integument (i.e., skin, hooves etc.) and damage to the respiratory system.

Feet with their sensitive tissues are particularly susceptible to fire, in both sheep and cattle. Veterinarians spoke frequently about the importance of assessing damage to the coronary band, the connection between the proximal hoof and skin of the leg. In cases of moderate pathology the coronary band may be mildly burned, but not severely enough to lead to separation of the skin of the leg and the hoof.

Skin burns were considered moderate when a small proportion of the body was burnt (e.g., <5–10%), and this was not generally full thickness. This frequently included damage to teats [also see (16)] and other exposed areas (e.g., vulva, inguinal and axillary areas). It is interesting to note damage to prepuce or scrotums was only rarely discussed, and this may reflect the relatively small proportion of entire male animals in Australian livestock populations in bushfire affected areas.

- Severe

Flocks of sheep could be found dead and packed together. Here sheep had presumably flocked together as the fire approached with sheep on the inside for the flock often suffocated and sheep on the outside dead from burns. In cattle this behavior was not reported, instead dead and burnt cattle were generally a smaller proportion of the herd and were dispersed across wider areas.

Severely affected stock included stock that were found dead with 100% skin burn coverage. However, some stock were still alive but comatose and recumbent even with extensive burns (e.g., 100%) to their bodies when visited one or more days after the fire had occurred.

Skin burns could be full thickness and across much of the body, so that the animal appeared charred. For example:

Veterinarian: “*There was one farm that we went to, that the biosecurity officer who was with me didn't realise that they were Herefords.”*
Interviewer: “*Oh he thought they were Angus, because they were black?”*
Veterinarian: “*Yes, it wasn't till we came across one that had calved post fire*.
*Yes, it had calved and we could see it was a dead Hereford calf.”* [IV3]

Other skin burns may be less than the whole body, but still extensive enough to lead to severe pain and suffering or later death as time passes.

Other stock that were severely affected included those with severe respiratory disease, such as with increased respiratory rates, exercise intolerance (hypoxia), open mouthed breathing and frothy nasal discharge. This indicates severe and acute respiratory pathology, presumably associated with burns to the respiratory system.

Hoof pathology was a particularly important area. A common pathological finding was that the coronary band had burned. This could present initially as a cracked or blistered coronary band before complete separation between the hoof and the skin of the leg as the coronary band split and lost integrity. This would often result in the hoof falling off the foot as it would lead to the coronary dermis separating from the overlying hoof. The sloughed hoof would appear normal and would leave exposed the underlying tissues of the hoof (e.g., underlying dermis, digital cushion and phalanx bones). These stock were extremely lame and were usually recumbent, or sometimes found in water bodies (dams) which the veterinarians interpreted as an attempt to relieve pain.

##### Time to Develop Pathology

Most informant veterinarians discussed that it takes several days for the full extent of burns to become evident. For example, on the day after a fire, burnt feet can appear relatively normal, with just an inflamed coronary band. However, over 3–4 days, if the burn is severe enough, the periople (coronary band) can split, the coronary dermis can release and the hoof can slough off. Likewise, skin burns on the body or extremities can appear relatively normal on the first day (for example a subtle leathering of the skin where skin loses its elasticity). However, after several days it can become an eschar (a necrotic slough of skin) and after several weeks be a large granuloma with skin migration from the edges (if the animal survives). For example:

Veterinarian: *And, actually, I saw a few cattle that I shot that would have been burnt three weeks before I got to them, by the time that I saw them. And so probably three weeks before they would not have looked that badly burnt. But when I got to them, they had sheets of skin hanging off them*.

Interviewer: *Muscles exposed, subcutaneous tissue?*

Veterinarian: *Yeah, Yeah, it was. It would have been the full amount of skin was hanging off them. So I would think that day one if I'd seen them, I probably would have thought that they were a mildly burnt animal, three weeks later there were sheets of skin hanging off them*. [IV2]

Other studies have also found that pathology develops over time, including over a period of days and weeks (12). It is noted that the prolonged time to development of visual gross pathology does not reflect the time for pain to be perceived by a burnt animal. For example, immediate pain may be associated with nociceptors being stimulated; animal burn models demonstrate that inflammation begins immediately as cells are injured as they release pro-inflammatory factors with inflammation also associated with pain (27). Bushfire injured livestock have demonstrated clinical signs consistent with pain after bushfire injury, for example inappetence, sternal recumbency and reluctance to move (12). Appropriate pain management should therefore be considered, even if early examination does not suggest severe injury.

#### Possible Treatment Outcomes

##### Strategic Options Available

Severely bushfire affected livestock often died on the farm before veterinary assessment. However, for livestock that are burned with less severity, or for well-resourced livestock owners who may be able to treat stock, management options are available. This section explores the several options informant veterinarians considered for burnt livestock, each with relative advantages and disadvantages. These include:

- Immediate euthanasia

A bushfire affected livestock animal is euthanized on the farm of origin, usually with a rifle shot to the brain.

This option is indicated for stock that are severely fire affected, where welfare is severely compromised and that cannot be successfully treated. In general, euthanasia was reported to be indicated when hooves had already sloughed (or were considered likely to slough in the near future) or where full thickness skin burns were evident across ~5–20% of the body or more and those stock were not fit to transport to an abattoir.

This was a common strategic option employed by veterinarians assessing moderately or severely fire affected livestock and is in-line with published government guidelines (28).

- Salvage slaughter

A bushfire-affected animal is immediately transported to an abattoir and slaughtered promptly on arrival under a commercial arrangement.

If an animal was bushfire-affected and required euthanasia (as above) but was fit for the intended journey (fit to load) under the Australian Animal Welfare Standards and Guidelines for the Land Transport of Livestock (29, 30), it could be slaughtered at an abattoir as an alternative to on-farm euthanasia. Briefly, a fit to load animal is one that can walk independently, is free from severe injury or distress and is strong enough to make the journey. An animal that is not fit to load would include where its condition is “likely to further compromise its welfare during transport” (29).

This was reported to have the advantage of being logistically more feasible (e.g., on-farm disposal of bodies is not required and response resources not allocated to destruction of livestock) and more acceptable for the producer as it returns some financial value from the slaughtered cattle to the producer.

However, salvage slaughter was used cautiously. Veterinarians considered it an ethical obligation to avoid undue suffering in injured livestock, and transporting an animal that is not fit to load is also legislated as an act of cruelty and liable to prosecution. Therefore, a conservative assessment of fitness to load was common in borderline cases. In addition, if there are obvious visible signs of burns on the animal, it is likely that these parts of the animal would be condemned, for example due to oedema in burnt areas (or the entire animal if the burns were extensive). This could make processing the animal financially unviable. A typical example of an animal that may qualify for salvage slaughter would be a cow with a burnt teats that would not have a productive future and that could be slaughtered promptly in a nearby abattoir with minimal carcass condemnations. Whilst a modestly injured animal like this may experience some welfare compromise on the journey (e.g., painful teats), decision making appeared to be a compromise between practicalities (ability to euthanize and dispose of bodies), animal welfare and financial compensation for the farmer (farmer welfare).

Particular care was taken by veterinarians to assess the hoof structure (to ensure that the hoof would remain intact during transport to the abattoir), that slaughter would be immediate (i.e., that the abattoir had availability to slaughter stock immediately) and that the journey length was suitable given the state of the animal, based on the veterinarian's assessment.

“[Salvage Slaughter] *would be OK if the animals could still walk onto a truck. And if udders were burnt to a point where they wouldn't be usable for breeding stock. I recommended straight to an abattoir*.

*There were a few where when I was looking at their feet, I felt like they were still lame, and I couldn't ethically feel comfortable them getting onto a truck. So they were given the option to treat or euthanise.”* [IV4]

Cooperation with livestock selling agents assisted the process, as they had contact with local abattoirs and could arrange a guaranteed processing slot. Livestock agents reportedly worked very hard for the clients, exhibiting bravery and altruism during the process of facilitating rapid salvage slaughter and minimize welfare impacts. As one veterinarian stated:

“*The agents were on the ground before we were…….. He did a phenomenal job, he is a hero.”* [IV3]

- Retain and cull later

Some stock had minor damage that could be treated on farm, with recovery expected without ongoing substantial animal welfare impacts. This may include those stock with burnt teat tips. These stock would likely have a poor productive future, and were often in sub-optimal body condition due to the drought that was concurrent with the fire season. However, keeping them on farm to recover for several months allowed these animals to put on condition, enabling improved carcass quality for commercial slaughter at a later date.

- No intervention

Whilst retaining burnt stock on farm with no treatment was not advocated by veterinarians, this sometimes occurred. This may have been because cattle disappeared into bushland (unrestrained by fences) or were not noticed or not considered by their traumatized owners. Some livestock did recover without intervention in cases reported by veterinarians although likely experienced a negative animal welfare state during their recovery.

- Treat on farm and normal productive capacity in the future

Some injured livestock could be retained on farm with appropriate veterinary and nursing care. They could then be expected to recover and have a normal productive life in the future. As an example, one producer opted to euthanize many of the ewes on her farm after a fire but retained most of the rams to conserve the genetic line of sheep. These rams had their burns bandaged and dressed and received appropriate food and medication (systemic antibiotics and pain relief) over a long period of time until their hooves grew back. Sperm motility was assessed post recovery to confirm that their long-term fertility was adequate.

- Hospitalization and intensive treatment

An alternative management pathway is to refer stock to a veterinary hospital for intensive treatment. However, in the bushfire affected regions there were very few large animal hospitals suitable for hospitalization of large animals and capacity was limited. In addition, transport was difficult and treatment would have been too expensive given the value of most of the animals that had been burnt. No sheep or cattle were reported hospitalized by informant veterinarians.

Some government veterinarians reported that a minority of local private veterinarians were occasionally treating stock in the field (*in situ* on a farm) that they deemed should have been euthanized for welfare reasons. The treatment provided was reportedly guided by treatment recommendations for other burnt livestock species (horses) in intensive care or hospital settings (31). However, the field treatment that occurred was not equivalent with the treatment reported in the publication. It is unclear if similar treatment was widespread, but if so, species-specific education about prognosis and treatment of badly burnt livestock and the need for immediate euthanasia may be required for some parts of the rural veterinary profession.

##### Medications and Treatments Used

Under the circumstances of limited access, emergency conditions and lack of access to veterinary hospitals, treatments used on livestock in the field were relatively rudimentary. Treatments that were practical and used or recommended by veterinarians included:

- Bandaging and dressing
- Systemic antibiotic use (especially with long-acting antibiotics such as oxytetracycline or penicillins)
- Non-steroidal anti-inflammatories (NSAIDs) such as meloxicam
- Topical treatments containing local anaesthetic, antiseptics and adrenaline such as off label use of Tri-Solfen® (https://apvma.gov.au/sites/default/files/publication/14121-prs-tri-solfen.pdf)
- Appropriate feed and water and general nursing care.

There was no widespread use of more complex and resource intensive burn treatments such as hospitalization with skin grafting or skin culture, fluid treatments or surgery (e.g., debridement).

#### Risk and Protective Factors for Bushfire Exposed Livestock Injury

Veterinarians provided opinions on what they thought were protective factors for bushfire-associated burns on livestock. These opinions allowed hypotheses to be developed about risk factors for burns.

It is important to note that these are anecdotal observations by veterinarians and could not be tested as formal risk factors in this qualitative study. However, a separate quantitative epidemiological study (a case-control study) where risk factors are formally tested against bushfire injury has been conducted concurrently as part of a broader research project (BC, Unpublished data).

##### Proactive Management Steps to Protect Stock

A key step taken that protected stock when fire arrived on farm was moving stock to a suitable location where bushfire was less likely to impact them. This may include regularly moving stock on a farm as fire fronts come from different directions. Suitable locations were generally on the same farm for larger producers but could be off-farm to nearby cattle holding infrastructure such as saleyards for smaller hobby farmers. As one veterinarian reported:

*She kept moving around it* [the fire]. *She had moved the animals multiple times and not one of her animals was affected, and she had quite a few stock*. [IV3]

A suitable location included:

- a dairy yard or cattle yard where cattle could be tightly held and where sprinklers could be activated.
- bare containment paddocks with very short grass that were away from woody vegetation.
- lush, irrigated paddocks with green grass.
- paddocks that were ploughed to remove grass in preparation of the fire.

In these locations, fire could not progress due to lack of fuel and hence stock were protected from burns.

A practical impediment to implementing this approach was having adequate notice of when a fire would arrive. As an example, one veterinarian noted what happened in a valley during a previous fire.

“*Actually, the Indigo Valley Fire that was a hot windy day, and fast-moving grass fire that started without any warning or anything like that. And the upper part of the fire, which is probably the area I spent most of my time in, there were quite a large number of properties with burnt stock up the valley because that's the way that the fire went. But as you moved up the valley, there were very few properties with burnt stock. And I think that the fire activity all the way through the valley probably would have been the same because the only thing that stopped the fire was it got to the end of the valley at the end of the day. And then there was a sort of a change in wind and a little bit of rain, and that kind of pulled it up*.

*But all that afternoon, the fire basically travelled up the valley at a similar sort of intensity. And so the only difference could be that the people that live near the start of the fire had less time to do something about preparing their stock from the ones further up the valley.”* [IV2]

This lack of warning was a particular concern in cross border areas where separate jurisdictional fire authorities were managing fire either side of the border. In Victoria in the upper Murray, farmers close to the NSW border did not receive warning of the fires approach and hence had less time to prepare for the fire.

Other protective management actions discussed included traditional fire management activities such as fire breaks, back burning, actively fighting fire with water and much earlier preparation with prescribed burning (e.g., previous year).

Providing stock access to paddocks without woody vegetation and with drainage lines and broad gullies (moist areas) where fire could pass over them were also reported to be protective.

##### Risk Factors for Livestock Burning

Unsurprisingly, the type, amount and proximity of vegetation around livestock were reported to be strongly influential risk factors to livestock being burnt. The proximity of woody vegetation, especially forested area was a strong indicator that the fire would be more intense, faster moving and riskier for livestock. A veterinarian reported that:

*Yeah. I mean, the properties where we euthanised everything were those bush blocks or where everything decided to run into the bush*. [IV2]

Landscapes with hills and uneven topography where fire could advance quickly uphill were risky. In addition these sort of areas, are harder to manage for fire risk (such as harder to control woody weed growth) and harder to muster stock from at short notice.

Another risk factor was where stock were trapped and could not maneuver around a fire. For example, gullies with thick blackberry infestations impeded escape, as did fences, and small paddocks.

##### Chance

Weather conditions were reported to be very influential on the fire intensity, and hence the risk to stock. A very hot, dry and windy day lead to more intense fires. Therefore, the weather conditions that coincided with a fire reaching a farm impacted the severity of stock damage, largely due to a complex interaction of proximity to fire, speed of fire, random events (e.g., spotting) and weather conditions. There is often no apparent predictable pattern to this interaction, so to some extent chance played a part in whether or not stock were injured. As one veterinarian noted about a farming area being threatened by fire:

“*They've got a range and the range kind of runs at the back of them and they would have been faced with fire. They must have had two or three weeks of fire. But I do think they were they were lucky. I think the whole time they had to be very alert. They were doing a lot of containment activity, and I think you (and not to say it wasn't stressful because it was constant for weeks on end) but it always just seem to be that as it kind of was getting closer, the wind would change, and then sort of push it away. Fire come down and then push away. And, you know, a lot of them were expecting at some point they're going to have a really bad day. So constantly alert to that very bad day, but they just didn't happen to get one of those days.”* [IV1]

### Analysis of the Decision-Making Framework Veterinarians Used to Decide What to Do With Bushfire-Injured Livestock

While there was little data provided by the veterinarians on the proportion of burnt stock that were treated and recovered, data on the proportion of dead stock that died of bushfire injuries was available from one district. Of these stock dead due to bushfires, it appeared that 12% of cattle and 23% of sheep were euthanized by government staff rather than dying from just fire injuries. This indicates that the decision to euthanize stock is an important decision for producers, and one largely made on recommendations of the attending government veterinarians. It is important because euthanizing livestock has financial and welfare impacts on farmers, but not euthanizing livestock can have welfare impacts for livestock. It is thus important to understand the decision-making process of attending veterinarians.

Theoretically there are several strategies that can be implemented to manage bushfire injured livestock. See section above—Strategic options available. However, in practice, two pathways were generally pursued, immediate euthanasia or retention and treatment on the farm, with salvage slaughter rarely deemed appropriate. Therefore, this section focuses on these two most common strategies.

Three themes were identified by most veterinarians that appear to explain how assessing veterinarians decided on a treatment strategy. These are:

- Pre-existing beliefs.
- Observations of pathology and clinical signs and level of prognostic doubt.
- Assumptions about the future.

They are described along with sub-themes in following sections.

#### Pre-existing Beliefs

This theme considers some beliefs and duties of attending veterinarians that may affect application of treatment strategies and that are independent of pathology. That is, it is not a simple matter of assessing the ability of a burnt animal to recover, rather it is also a complex decision based on non-biological factors associated with clinical training, societal and ethical beliefs, and experience.

##### Welfare

Veterinarians felt ethically that euthanasia was the best course to pursue for severely bushfire-injured affected livestock, even if they could survive. As one veterinarian stated:

“*If there was anything, if I thought something was unlikely to survive, I really encourage people to use euthanasia. I think that greatest gift to give. We couldn't have something standing there suffering.”* [IV6]

No definition of suffering was provided by interviewed veterinarians. However, many veterinarians reported that livestock owners felt the same way. This indicates that the welfare of animals is a broader societal consideration.

For example:

*A lot of the euthanasia happened before we could get out there, by local people*. [IV2]

However, a key issue was that non-veterinarians involved with assessing stock (for example a farmer assessing their own livestock) were perceived to tend toward under-estimating the severity of the injuries in the early stages. These individuals would not realize the severity of injuries that would likely develop over time as injuries became more apparent (e.g., as skin sloughed etc.).

In summary, despite many stock possibly being able to survive fires, veterinarians often recommended euthanasia:

“*There were plenty I think that got burnt and significantly badly burned that would have survived, but it just would have been awful.”* [IV6]

#### Observations of Pathology and Clinical Signs and Level of Prognostic Doubt

##### Decision Points (Criteria for Euthanasia Verse Treatment)

Veterinarians discussed what they thought were some clinical criteria that could be used to decide whether to treat or euthanize bushfire affected livestock. These include:

- Hooves:

Where the examination occurred quickly after a fire (e.g., 1 day after the fire) and before full progression of pathology, the key criterion was damage to the coronary band. Euthanasia was recommended if the damage was significant enough that it could or would lead to separation of the hoof and skin of leg and later sloughing of the hoof. Significant damage included cracking and severe burns or inflammation of the coronary band. As most examinations occurred quickly after fires (e.g., within 1–2 days of injury) before full development of pathology, to some this assessment relied on experience and what may happen to the hoof in the future. Experience often included a veterinarian's previous experience treating bushfire affected livestock or advice from more senior veterinarians. That is, prognostic or predictive thinking was employed to determine how the pathology (especially separation of the hoof) may develop and guide advice for euthanasia.

If pathology had time to progress before examination (i.e., examined after 3–4 days) then the hoofs attachment to the foot was the key criteria. In general, movement of the hoof relative to the foot was an indication that it was likely the hoof would slough off.

In more severe cases where hooves had sloughed off, this was an indication for immediate euthanasia.

In summary, all veterinarians were aware that there would be a progression of clinical signs over time. However, there was doubt in some circumstances as to how bad the pathology may be once time had passed (i.e., how the hoof burns would progress). In general, it appeared a precautionary principle was applied and it was assumed that severe burns to the coronary band would lead to hoof sloughing and thus livestock should be euthanized. It appeared that this assumption about future pathology was a critical decision point, but these decisions had to be made without a clear prognostic indicator or without certainty by the assessing veterinarians in some instances.

- Skin burns (depth and thickness)

Several veterinarians reported they used existing departmental guides for euthanasia based on skin burns. For example if 5–10% or more of an animal was burnt to full skin thickness then they would recommend euthanasia. However, the reported range that lead to euthanasia in the 2019/2020 bushfires varied from 5 to 20%. And it was clear that many veterinarians saw animals that survived with significantly greater proportions of the body burnt (e.g., 50%). Several veterinarians exceeded the available guidelines of proportion of body burnt based on their clinical judgement of the animal's welfare compromise and capacity to recover, especially if there was no insurance coverage in place for bushfire affected livestock. This presented veterinarians with an ethical dilemma, to find the right balance between their professional obligation to prevent pain and suffering in animals, yet to also protect the financial viability and welfare of the farmers which they assist.

The thickness of the skin burn was also important. Superficial burns were viewed more favourably, but if the burns were full thickness then it was considered more conservatively (i.e., were more likely to be culled).

Although the interaction between size of burn and depth of burn was important, there was no simple criteria for culling reported that combined both depth and area of burn.

##### Biological Ability to Recover From Burns Can Be High

Very severely burnt animals often died at the time of the fires or shortly after. However, there were a proportion of substantially burnt animals that were not assessed and euthanized at the time of the fires. Instead they survived bushfire burns as evidenced by being presented for examination for the first time many weeks after the fires. This indicates that many stock could potentially recover from bushfire burns, even if they were severe.

“*I saw one cow that I saw three weeks after the fire. She had been trucked elsewhere, shouldn't have but she was, three weeks after the fire and she had scarring that it was quite obvious that she'd had deep burns to one side of her body. Probably 50%. Was healing amazingly. I think it would have been full thickness. It had started granulating and was starting to contract already and come in from the sides. ……*

*And I think that animal, if I'd seen her on the day of impact, I probably would have euthansed. It was an interesting moment to me to go, well, actually, they can heal. And I suppose that's true of burns, but maybe not so much on feet, but on skin, so long as they don't get infected, they will heal.”* [IV1]

Similar observations were reported by several of the veterinarians interviewed.

Thus, it is apparent that many badly burnt animals do have a biological ability to recover, especially from extensive skin burns. However, in most cases where these cattle were observed shortly after being burnt, assessing veterinarians would elect to euthanize stock, rather than treat stock.

##### Experience and Information Sources for Attending Veterinarians

Whilst veterinarians were experienced in rural practice, competent and capable, several reported they had little experience with bushfire affected livestock and in recognizing the pathology of bushfire burns. That is, several veterinarians were attending their first fires. Their sources of information on prognosis were limited to Government Departmental guides on treatment of bushfire affected livestock and discussions with more experienced veterinarians. Whilst Departmental guides are useful documents, they are based on a small number of contributing individuals and anecdotal experience with little research base behind them. Similarly the same sort of information was provided by experienced colleagues who also could not generally attend fires with veterinarians due to resource constraints.

Thus, some veterinarians were making decisions with only a theoretical understanding of bushfire affected stock, and no practical experience of prognosis. This tended to lead veterinarians to euthanize livestock as a precautionary measure to avoid possible adverse welfare outcomes. With greater experience in future, several veterinarians may not have recommended as high a proportion of livestock be euthanized. For example, one veterinarian spoke about relying on Departmental guides until they were experienced and then using their own experience in part to guide prognosis and decision making by more refined categorization of affected livestock:

*I looked at a DPI document. You know, maybe DPI was suggesting, if you've got more than 5 to 10% of the cow burnt, then you had to cull it for a full thickness burn. Particularly if it's a full thickness burn. Yes, the full thickness burns were covering one side of the cow, so I deem that 50% of full thickness. So you're a goner no matter whether or not you're a human in intensive care, hospital bed or you're a cow*.

*And then I suppose the further into it I got, maybe the more lenient I became. But you think you know, yet the more you see, the more you start to try and sort of get your category and then you start to unconsciously put animals on the scale within that category. I always end up doing the same with welfare cases as well. You know, you've got a high risk one animal, and then you've got is not quite as bad or worse, but it's not a high risk two, so yeah, scale around that whether they are going to get culled or not*. [IV5]

*Summary of theme* Defined pathological indicators of when burnt livestock should be euthanized were useful, although the application of prognostic indicators was complicated by the gradual progression of signs over several days. That is, pathology may be very subtle in the first days after fire exposure and livestock may not be clearly identified as being in a severe category initially. In addition, some attending veterinarians were unavoidably inexperienced at assessing burnt livestock (as fires are rare), although their experience increased rapidly over time. These factors led to some uncertainty on the prognosis for some burnt livestock. Where uncertainty occurred, in some instances veterinarians culled some livestock on a precautionary basis. Clearly, as many livestock can survive with severe burns, some culling that occurred was for reasons other than a biological ability to recover (see next themes below).

#### Assumptions About the Future

In deciding on a treatment pathway various practical considerations were relevant to veterinarians. Mostly these were inferences about the future, made by attending veterinarians.

##### Costs and Resources (Including Human) to Treat

In general veterinarians believed intensive treatment of animals was impractical for many bushfire-affected livestock and instead chose euthanasia for stock that may otherwise have recovered with intensive treatment. That is, they were seeking to avoid incurring future human and financial costs on behalf of producers.

Successful treatment of burnt stock is often time-consuming and requires great effort, and most livestock owners had many responsibilities and difficulties after the fires. That is, they may have had deceased family members, their house burnt down, extensive infrastructure damage on their farm, access to services impeded (e.g., veterinary services) and financial losses. This practically limited the time that livestock owners could spend on intensive nursing of bushfire affected stock. Without time, money and the mental resources available to conduct the required treatments, then the probability of appropriate nursing and treatment of stock was reduced, and the alternative was euthanasia of livestock. For example as one veterinarian stated:

*Absolutely, some were keen as mustard to treat, whatever needed to be done. So we threw the book at it. A couple of the cattle people well, they were not interested, like they had lost half the sheds, the house, they were, you know, more concerned with the fact they were alive. And, you know maybe getting some hay to the ones that were alive*. [IV5]

The successful treatment of bushfire-affected livestock is complex and highly skilled but little researched with most information available for the treatment of general burns in other species such as companion animals and horses (31–35). Most veterinarians assessing livestock were government veterinarians and had the skills. However, the general policy for the organizations employing these veterinarians was that they do not offer medicines and treatments to livestock, instead they simply assess, advise on treatment and assist in euthanasia of stock. Medicines and treatments are instead offered by private veterinarians, which are typically an expense to a livestock owner and are not always accessible after a fire. Thus, lack of access or ability to pay for veterinary services tended to be an impediment to treatment of livestock, if not to assessment and euthanasia.

Notwithstanding this, there were rare reports of successful treatment of livestock. For example, in one instance a veterinary nurse was able to stay at a bushfire affected farm for weeks and nurse genetically important rams who eventually recovered and were subsequently fertile. This was summarized by a veterinarian who observed the treatment:

*And I mean, the thing that helped with that was that she had a vet nurse friend who stayed with her, who did all the treatments. Managed to get free antibiotics or donated and got some pain relief and was able to change bandages and things. And we saw sheep in that that you know, they did slough their hooves, but they were managed with bandaging. And they actually regrew those hooves. So you know it is possible to do it. But its very time consuming and very expensive*. [IV7]

##### Veterinary Access to Farms

During the fires, access to fire affected farms was relatively limited due to safety restrictions, and veterinary resources were stretched due to the number of affected farms in each fire-affected region. This meant that veterinarians tried to limit visits to a single visit per farm for pragmatic reasons, as future access and resources to attend the farm on multiple occasions may be limited. Sometimes this was not the case, with some farms receiving multiple visits when possible and required. In addition, veterinarians tried to reach farms within the first day after a fire to optimize welfare outcomes (reducing the possibility of negative welfare states while awaiting veterinary assessment) for fire affected livestock. That is, if there were livestock requiring euthanasia, it was better to do this as soon as possible to reduce any possible livestock suffering. For example, an interviewed veterinarian stated:

*To me that was drawing out a very long, painful process. I guess my theory was go hard, go early and then have that job done to, like, don't have to keep going back for return visits*. [IV2]

However, due to the time taken for the worst pathology to develop, this meant that with a single and early visit, decision making on what to do with animals was based on somewhat incomplete information. This is explored further below.

##### Attitude and Ability of Owners

Several veterinarians reported the attitude, resilience and ability of livestock owners to provide the care required for successful treatment was a criterion they used to decide on whether treatment should be pursued or whether livestock should be euthanized.

For example:

“*It's a little bit of summing up what the producers are like? What they're going to be able to manage how well you think that they're going to be able to do it plus how much they know about animals and treatment alone? But I tend to take a fairly hard approach. It's a bit of a case of if in doubt, take it out on the first day.”* [IV2]

As a further example from another veterinarian:

“*….like if they were too traumatised, they couldn't help themselves let alone animals.”* [IV5]

##### Practical Considerations

Veterinarians considered various practical features in any given scenario, to determine whether treatment was likely to be possible the subsequent days or weeks. For example, many farms lost cattle yards when they burnt and had no ability to physically yard and treat injured livestock adequately. This meant that euthanasia for severely affected stock was a more practical solution.

Other practical considerations include whether owners of livestock had insurance policies for burnt livestock. Euthanasia was more likely if livestock owners had insurance for affected livestock as it reduced the financial impact on the livestock owner. Several veterinarians mentioned insurance as an influential aspect to decision making.

“*Although it probably shouldn't make a difference, but it's always a little bit easier to do that when you ask the owner of the animal if the stock are insured.”* [IV2]

#### Summary of Thematic Analysis: Conservative Decision Making

There were complex factors impacting decision making about how to manage burnt livestock.

While welfare was one of the primary considerations, it appeared that there was at times conservative decision making when deciding how to treat moderately or severely affected livestock. Treatment tended to euthanasia as veterinarians were risk averse (e.g., welfare) and had various practical considerations to take into account. Many stock may have survived their bushfire injuries but for complex reasons (especially welfare) they were instead euthanized without treatment being attempted.

In particular, complex interconnected issues of gradual progression of pathology with early assessment of stock after being burnt, limited access (i.e., difficulty re-attending stock), limited veterinary resources, professional desire and responsibilities to alleviate suffering caused conservative decision making by assessing veterinarians. For example, a veterinarian who visited a farm and saw stock that may be able to be retained on the farm with treatment, but where there was a risk of further decline in their clinical status, were sometimes more likely to euthanize the livestock in question, rather than recommend treatment of the stock and observe what happens over succeeding days. Perhaps this could be phrased as the assessing veterinarians applied a precautionary welfare principle and euthanized stock early in the progression of pathology. This likely lead to some limited excess euthanasia of livestock, similar to findings from previous research (18), but reduced welfare impacts and enabled pragmatic resource allocation.

As one veterinarian stated:

“*I guess my theory was go hard, go early.”* [IV6]

#### Decision Making Model

**Figure 2:**
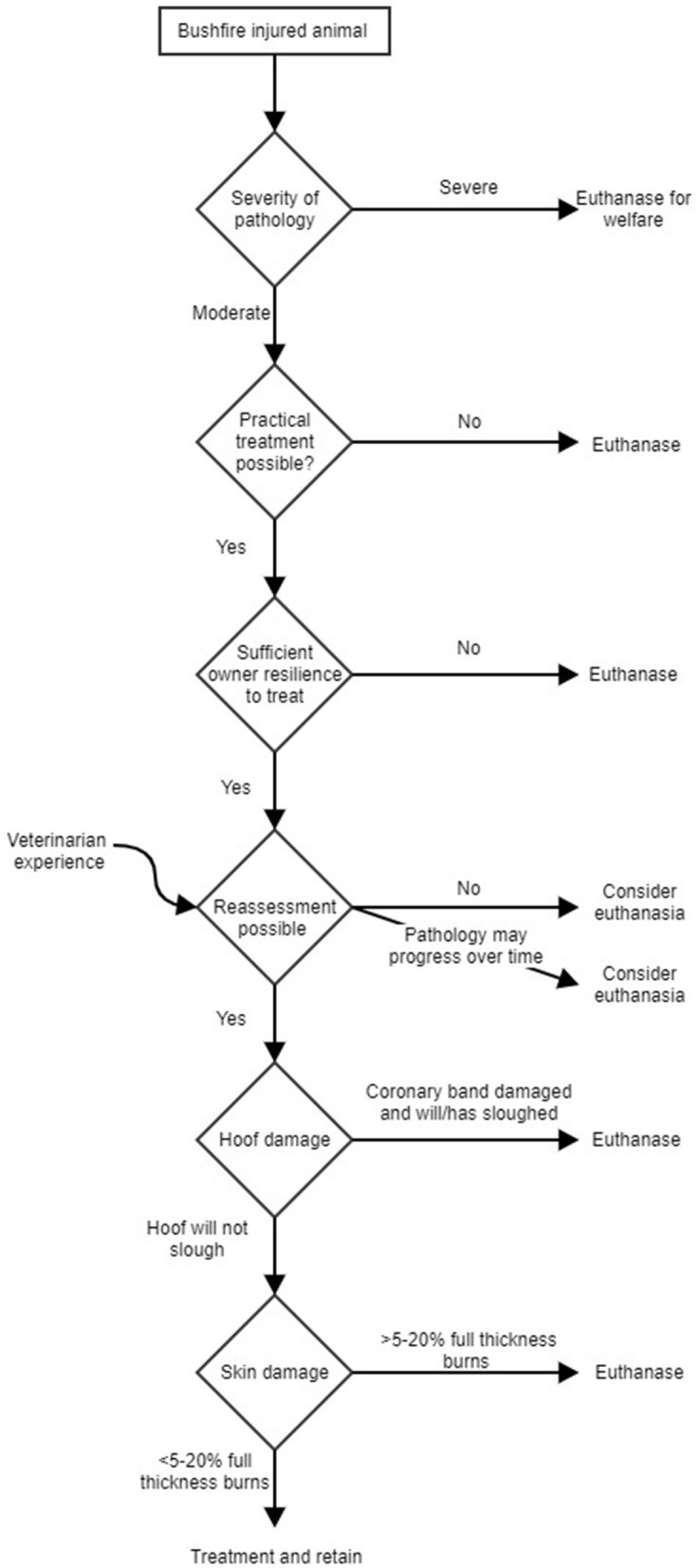
A decision making process for treatment or euthanasia by assessing veterinarians attending moderately or severely bushfire injured livestock.

Whilst there are several strategic treatment options available for bushfire injured livestock (see section “Strategic Options Available”), there were two main options employed in most situations by most veterinarians: Immediate euthanasia or treat and retain on the farm (either retention for normal production or later culling). We present a simple model that represents the decision-making process of the majority of veterinarians that were interviewed for these two options (treat or euthanasia). See Figure 2 for a summary, but the decision-making pathway is outlined in text below.

The first consideration when deciding to euthanize or treat was the severity of pathology. Mildly injured animals are not considered in the model as they were generally treated simply on farm and retained. Severely burnt animals were euthanized immediately. The difficult decision point was if an animal was moderately injured. If the animal had moderate burns then the animal could be considered for treatment and retention or euthanasia on the farm. However, even moderately burnt livestock would be euthanized if the owners did not have sufficient personal resources (emotional, time or money) or practical resources (e.g., yards) to treat the animal and relieve suffering.

An important consideration was whether assessing veterinarians had the ability to re-attend and reassess moderately burnt livestock following an early visit, early during the development of pathology. That is, if there was uncertainty about the progression of pathology (e.g., marginal cases) and veterinarians could not re-attend to re-assess, then cautious decision making about leaving stock alive and injured were made with veterinarians more likely to recommend euthanasia. To some extent veterinarian experience made a difference here. Inexperienced vets that were uncertain of progress were more likely to recommend euthanasia in the face of uncertainty to mitigate the risk of subsequent welfare issues.

However, after these considerations, for moderately burnt livestock, the key considerations were the extent of feet damage and the extent and depth of skin burns.

## Discussion

This research, which both describes current practices in management of bushfire-injured livestock and analyses the decision-making behind these practices, has illuminated challenges in the immediate post-fire period. Key areas identified include effective prognostic assessment for livestock as well as how to make difficult decisions to address compromised animal welfare while balancing the needs of the broader farm system that these animals exist within. With climate change associated with increased incidence of bushfires (7, 8), this study makes an important contribution to the presently sparse research into bushfire impacts on livestock, needed if the effects of bushfires on livestock are to be ameliorated in the future. This research also provides some hypotheses about the risk factors for livestock injury during bushfires that could be investigated further.

It is also important to realize that many burnt livestock have a biological ability to survive with severe injury, as evidenced by stock surviving and healing with no assessment and treatment for many weeks after fires had occurred. For example, some livestock with significant portions of their bodies burnt to full thickness were observed by veterinarians for the first time many weeks after fires. This highlights the need to make decisions about euthanasia on welfare grounds for severely injured animals, not on the ability of an animal to survive, as undoubtedly a proportion *can* survive severe injuries. An ethical dilemma arising for those assessing the livestock is: under what circumstances it is reasonably to allow an animal to recover rather than be destroyed, and what measures (such as nursing treatment and appropriate pain relief) are necessary to mitigate animal welfare compromise. Further investigation of this dilemma may be helpful but was beyond the scope of the present study.

The decision on whether to treat or euthanize moderately bushfire injured livestock was generally a difficult one with impacts on the farmers who owned the stock including severe financial loss. However, injured livestock that are suffering excessively, that cannot for practical reasons receive analgesia, or are unlikely to recover require euthanasia for welfare reasons. It is important to note that there we are aware of no present research directly investigating managing livestock pain associated with burns. In this study, the main pain relief available for prescription were NSAIDs, and off-label use of topical local anaesthetics designed for routine husbandry procedures. There were no instances of the use of opioid analgesics, which are indicated for analgesia of severe burns in human and veterinary medicine (32). Further research into the suitability and practicality of analgesics suitable for livestock with burn injuries that are intended for human consumption, including the importance of pain relief for even mild burn injuries, would be beneficial to guide prescribing in emergency conditions.

Consistent with other literature (18), assessment of livestock and subsequent recommendations for slaughter were at times conservative, resulting in a limited excess euthanasia of livestock. This is not a reflection of the assessing veterinarians who we interviewed, who appeared highly skilled, professional and compassionate. Instead, in some cases euthanasia may be associated with resource constraints, limitations in prognostic assessment and the likelihood of progression of bushfire injuries over time, and the need to avoid welfare impacts on livestock. Stock assessments occur rapidly after fires to ensure that severely burnt livestock can be euthanized immediately. However, for livestock that are injured moderately, at this early stage it may not be apparent if their clinical condition will progress to meet the criteria for immediate euthanasia or not. At this time point, burn injuries may not have progressed to their worse clinical severity. Concurrently, many farms and livestock need assessing and access is limited and dangerous, or not prioritized by emergency authorities. In these circumstances, veterinarians may need to limit the number of visits to any single fire-affected farm. For these reasons, it appeared that if there was uncertainty about the severity of injuries, a precautionary decision to euthanize stock immediately was sometimes taken, to avoid leaving livestock alive with progressing injuries that will lead to adverse welfare outcomes over time. Farmers were often traumatized at this time and may not always be in a position to objectively discuss decision making.

Examining the decision making that occurred (Figure 2), the main decision node that can be modified is the re-assessment node. That is, veterinarians may be able to assess the need for euthanasia more accurately if they were able to re-attend these animals and ensure that they were not worsening to an unacceptable clinical state. The solution may therefore be to provide additional veterinary resources and prioritize veterinary access to farms to enable sufficient repeat visits to allow additional time points to assess pathology and its effect on animal welfare. However, practical difficulties will persist, including access remaining difficult and dangerous, veterinary resources frequently constrained, treatments expensive and labour-intensive, and livestock owners still in a traumatized state. The option of revisiting to reassess pathology before euthanasia is only indicated where adequate nursing care, especially appropriate pain relief and the labor required to administer it, is available for injured animals in the interim.

The apparent absence of a modern burn classification system for livestock, such as is available in small animals (35) can limit communication and assessment of livestock. For example, the communication of burns by veterinarians in the interviews was limited to full thickness and partial thickness burns. However, in small animals, burns have been divided into a much more granular classification system, including superficial, superficial partial thickness, deep partial thickness and full thickness with a key for dermal layers affected, wound characteristics and healing. The development of a more granular classification system for livestock, that was linked to healing or prognosis and welfare would be an important tool that could be used by veterinarians when assessing bushfire affected livestock. Despite this, the application of such a tool may be limited on some farms where the ability to closely assess stock is limited due to damaged stock handling facilities.

Risk factors discussed by the veterinarians that affected the presence or severity of bushfire injury included proximity to woody vegetation (such as forest) and features which tended to trap stock in front of a fire (such as fences or blackberry infestations in gullies). Chance also played a part, with the interaction of when a fire reached a farm and the severity of weather conditions at that time having a major influence on fire intensity and risk of injury to livestock.

Conversely, there were several features that assessing veterinarians hypothesized could protect livestock from injury, based on their observations. One of the most important features was an adequate warning time of an impending fire. Where the warning was adequate, farmers could move stock to safer areas thus protecting them from fire, or implement fire-fighting actions. Safer areas for smaller producers were areas off farm, away from the fire. For larger producers, open paddocks away from woody vegetation with short grass and/or water bodies, or containing stock in dairy yards or cattle yards, were protective. However, many farmers were not perceived to have been able to respond to this type of early warning of an approaching fire, sufficient to enable livestock to be moved to safer areas. Whilst some late warning times were due to the nature of the fire (sudden and unexpected), in other cases, fire warning systems were inaccurate or slow or did not assimilate information from nearby fires across jurisdictional borders. This was also reported by the Australian Royal Commission into National Natural Disaster Arrangements (36). Therefore, faster warning times with accurate data, including across jurisdictional borders are urgently needed during bushfires to enable producers to protect stock. Improvements to these systems have been recommended in recent bushfire inquiries (37). These recommendations, if implemented will also assist farmers with response to fires by enabling livestock protection from burns. Other protective factors reported included active fire-fighting approaches, such as establishing firebreaks before a fire, fighting fire with back-burning or water, or defending stock with sprinklers and fire hoses.

In addition to burns to hooves, skin and other structures such as teats, acute respiratory disease was recognized by these veterinarians which may have important effects on both animal welfare and prognosis in affected individuals. Published peer-reviewed research on livestock pathology and injuries due to fire is presently extremely limited. This is an important gap although unsurprising given the emergency that bushfires present, where priorities appropriately include protection of life and assets, preservation of remaining livestock and emergency management of the welfare of injured livestock, rather than conduct of research. These limitations informed the qualitative design of the present study, which aimed to collect detailed and useful data after the emergency (and the timeframe for meaningful data collection from carcasses) had ceased. Importantly, of all the veterinarians interviewed, some having attended fires annually for 20 years, none had ever had the opportunity to conduct a formal post-mortem examination of fire-injured livestock, for example to examine respiratory system damage. Such basic information may provide important insights into prognosis and possible treatments, and while we hope this can be investigated in future, it would only be practical where veterinary resources were in excess to immediate emergency response requirements.

Finally, it is important to consider this research in the context of Australia's livestock population. Despite very widespread fires, the number of livestock directly fire-injured was surprisingly modest. Even in a severely fire-affected district, where many farms were subject to bushfire, only 7% of cattle were killed. Whilst these losses are significant, especially to individual farmers, it is not likely to lead to a large decline in the national herd. The widespread drought that preceeded these fires are likely to have had more significant impacts on the national herd due to increased selling of stock and poor reproductive rates.

In conclusion, this research suggests that most bushfire injuries in livestock were associated with burns to the common integument (especially hooves, but also skin and associated structures such as teats), although acute respiratory disease was also recognized. Based on veterinarian-reported risk factors, key actions to protect livestock from bushfires could include earlier and better warnings about where fires are so that stock can be moved to protected locations, protecting livestock with active firefighting techniques and management of fuel loads to reduce the intensity of fire. However, the unpredictability of when exactly fire will reach a farm and the severity of weather conditions at that time appeared to influence livestock injury risks, rendering the idea of preventing all fire-injury to livestock very unlikely. Veterinary decisions for managing moderately injured livestock were complex and at times uncertain, potentially leading to precautionary culling where prognostic assessment was uncertain. Prioritizing regular re-examinations of livestock after bushfire injury may reduce unnecessary euthanasia, provided appropriate nursing care including pain relief can be provided.

## Data Availability Statement

The raw data supporting the conclusions of this article will be made available by the authors, without undue reservation.

## Ethics Statement

The studies involving human participants were reviewed and approved by the University of Melbourne's Human Research Ethics Committee (Ethics ID 2057893.1). Written informed consent for participation was not required for this study in accordance with the national legislation and the institutional requirements.

## Author Contributions

BC, MW, and MB-T contributed to the conception and design of the study. BC, JC, HC, and MD collected data. BC analyzed the data and wrote the first draft of the manuscript. CP, MB-T, and MW made strategic comments on the analysis and manuscript design. All authors contributed to manuscript revision and read and approved the submitted version.

## Funding

This research was funded by Meat & Livestock Australia (MLA grant number: B.AHE.2102) and the Commonwealth Government of Australia and we acknowledge that funding.

## Conflict of Interest

BC, MB-T, and AH are employed by the company Ausvet Pty Ltd. The remaining authors declare that the research was conducted in the absence of any commercial or financial relationships that could be construed as a potential conflict of interest.

## Publisher's Note

All claims expressed in this article are solely those of the authors and do not necessarily represent those of their affiliated organizations, or those of the publisher, the editors and the reviewers. Any product that may be evaluated in this article, or claim that may be made by its manufacturer, is not guaranteed or endorsed by the publisher.

## References

[1] Filkov AI Ngo T Matthews S Telfer S Penman TD. Impact of Australia's catastrophic 2019/20 bushfire season on communities and environment. Retrospective analysis and current trends. J Saf Sci Resil. (2020) 1:44–56. 10.1016/j.jnlssr.2020.06.009

[2] Richards L Brew N Smith L. 2019−20 Australian bushfires—frequently asked questions: a quick guide. In: Library P, editor Canberra, ACT: Department of Parliamentary Services, Parliament of Australia (2020). Available online at: https://parlinfo.aph.gov.au/parlInfo/download/library/prspub/7234762/

[3] Borchers Arriagada N Palmer AJ Bowman DM Morgan GG Jalaludin BB Johnston FH. Unprecedented smoke-related health burden associated with the 2019–20 bushfires in eastern Australia. Med J Australia. (2020) 213:282–3. 10.5694/mja2.50545 32162689

[4] Kotsios N Twomey S. National livestock toll from raging bushfires mounts. The Weekly Times. (2020).

[5] Anon. 1270. 0.55.003 - Australian Statistical Geography Standard (ASGS): Volume 3 - Non ABS Structures, July 2016. In: Statistics ABO, editor. Canberra, ACT (2016).

[6] Anon. 7121.0 - Agricultural Commodities, Australia, 2017-18. In: Statistics ABO, editor. Canberra, ACT (2019).

[7] Flannigan MD Krawchuk MA de Groot WJ Wotton BM Gowman LM. Implications of changing climate for global wildland fire. Int J Wildland Fire. (2009) 18:483–507. 10.1071/WF0818728948418

[8] Liu Y Stanturf J Goodrick S. Trends in global wildfire potential in a changing climate. For Ecol Manage. (2010) 259:685–97. 10.1016/j.foreco.2009.09.002

[9] Gee C editor. Involvement of veterinary inspectors in bushfire situations. In: Veterinary Inspectors of NSW 1986 Conference Proceedings 69th Annual conference 1986. Sydney (1986).

[10] Dieckmann HG Costa LRR Martínez-López B Madigan JE. Disaster Medicine: implementation of an animal health database in response to the 2018 California Camp Fire. J Am Vet Med Assoc. (2020) 256:1005–10. 10.2460/javma.256.9.1005 32301654

[11] Malmo J editor. Assessment of cattle burnt in bushfires. In: Australian Cattle Veterinarians and Australian Sheep Veterinarians (ACV/ASV) Annual Conference. Hobart, TAS: Australia Australian Veterinary Association (2015).

[12] McAuliffe PR Hucker DA Marshall AN. Establishing prognosis for fire damaged sheep. Aust Vet J. (1980) 56:123–32. 10.1111/j.1751-0813.1980.tb05648.x 7436905

[13] McAuliffe PR Hucker DA. Recovery of sheep from bushfire damage. Victorian Vet Proc. (1978) 36:40–1.

[14] Morita N Traber MG Enkhbaatar P Westphal M Murakami K Leonard SW. Aerosolized alpha-tocopherol ameliorates acute lung injury following combined burn and smoke inhalation injury in sheep. Shock. (2006). 25:277–82. 10.1097/01.shk.0000208805.23182.a7 16552360

[15] Morton JM Fitzpatrick DH Morris DC White MB. Teat burns in dairy cattle the prognosis and effect of treatment. Aust Vet J. (1987) 64:69–73. 10.1111/j.1751-0813.1987.tb09617.x 3579751

[16] Prat NJ Herzig MC Kreyer S Montgomery RK Parida BK Linden K. Platelet and coagulation function before and after burn and smoke inhalation injury in sheep. J Trauma Acute Care Surg. (2017) 83:S59. 10.1097/TA.0000000000001472 28452873

[17] Rethorst DN Spare RK Kellenberger JL. Wildfire response in range cattle. Vet Clin N Am Food Anim Pract. (2018) 34:281–8. 10.1016/j.cvfa.2018.02.004 29935717

[18] Rogers J Scholz T Gillen A. Dealing with livestock affected by the 2014 bushfires in South Australia: decision-making and recovery. Australian J Emerg Manag. (2015) 30:4. 10.3316/agispt.2015280

[19] Smith B Taylor M Thompson K. Risk perception, preparedness and response of livestock producers to bushfires: A South Australian case study. Australian J Emerg Manag. (2015) 30:38.

[20] Traber MG Shimoda K Murakami K Leonard SW Enkhbaatar P Traber LD. Burn and smoke inhalation injury in sheep depletes vitamin E: Kinetic studies using deuterated tocopherols. Free Radic Biol Med. (2007) 42:1421–9. 10.1016/j.freeradbiomed.2007.01.041 17395015 PMC1899466

[21] Willson RL. Assessment of bush fire damage to stock. Aust Vet J. (1966) 42:101–3. 10.1111/j.1751-0813.1966.tb07630.x 6006007

[22] Wolff PL editor. Small ruminants in disasters - hurricanes to wildfires 2009. In: The North American Veterinary Conference. Gainesville, FL (2009).

[23] May C. Discovering new areas of veterinary science through qualitative research interviews: introductory concepts for veterinarians. Aust Vet J. (2018) 96:278–84. 10.1111/avj.12718 30129033

[24] Tong A Sainsbury P Craig J. Consolidated criteria for reporting qualitative research (COREQ): a 32-item checklist for interviews and focus groups. Int J Qual Health Care. (2007) 19:349–57. 10.1093/intqhc/mzm042 17872937

[25] Braun V Clarke V. Using thematic analysis in psychology. Qual Res Psychol. (2006) 3:77–101. 10.1191/1478088706qp063oa32100154

[26] Dyce K Sack W Wensing C. Text Book of Veterinary Anatomy. Pedersen D editor. Philadelphia, PA: WB Saunders (1987). P. 798.

[27] Abdullahi A Amini-Nik S Jeschke MG. Animal models in burn research. Cell Mol Life Sci. (2014) 71:3241–55. 10.1007/s00018-014-1612-5 24714880 PMC4134422

[28] Anon. Assessing Cattle After a Bushfire. Melbourne, VIC: Agriculture Victoria (2021). Available online at : https://agriculture.vic.gov.au/farm-management/emergency-management/bushfires/what-to-do-after-a-bushfire/assessing-cattle-after-a-bushfire (accessed December 9, 2021).

[29] Anon. Land transport of livestocK. Australian Animal Welfare Strategy (2012).

[30] MLA. Is the Animal Fit to Load? A National Guide to the Pre-transport Selection and Management of Livestock. Sydney, NSW: Australia Meat and Livestock Australia (2019).

[31] Herbert EW. Findings and strategies for treating horses injured in open range fires. Equine Vet Educ. (2018) 30:177–86. 10.1111/eve.12806

[32] Butkus CE Peyton JL Heeren AJ Clifford DL. Prevalence, treatment, and survival of burned wildlife presenting to rehabilitation facilities from 2015 to 2018. J Zoo Wildl Med. (2021) 52:555–63. 10.1638/2020-0093 34130398

[33] Vigani A Culler CA. Systemic and local management of burn wounds. veterinary clinics of North America: Small Anim Pract. (2017). 47:1149–63. 10.1016/j.cvsm.2017.06.003 28802983

[34] Hanson RR. Management of burn injuries in the horse. Vet Clin N Am Equine Pract. (2005) 21:105–23. 10.1016/j.cveq.2004.11.006 15691603

[35] Vaughn L Beckel N Walters P. Severe burn injury, burn shock, and smoke inhalation injury in small animals. Part 2: diagnosis, therapy, complications, and prognosis. J Vet Emerg Crit Care. (2012) 22:187–200. 10.1111/j.1476-4431.2012.00728.x 23016810

[36] Binskin M Bennett A Macintosh A. Royal Commission into National Natural Disaster Arrangements Report. Canberra, ACT: Australia Commonwealth of Australia (2020). p. 594.

[37] Owens D O'Kane M. Final Report of the NSW Bushfire Inquiry. Sydney, NSW: New South Wales Government (2020). p. 436.

