# Supplementary material for: The Australian 2019/2020 Black Summer Bushfires: Analysis of the Pathology, Treatment Strategies and Decision Making About Burnt Livestock
Source: Front Vet Sci. 2022 Feb 15;9:790556. doi: 10.3389/fvets.2022.790556 (PMC8886614; doi:10.3389/fvets.2022.790556)
Supplement: Supplementary file 1 [file Data_Sheet_1.docx]

Supplementary Material

# Interview guide used when interviewing veterinarians

- Introduction
  - Years of experience in rural practice
  - How many times (seasons) have you responded to fires?
  - How many farms did you visit in 2019/2020 fires?
  - Do they have your own stock or farms and was it bushfire affected?
- Pathology section
  - When you observed burnt livestock, what pathology did you see?
    - interested in anatomical area of animal that was burnt
    - internal (lungs) or external burns
    - thickness (i.e. full skin thickness, superficial etc.)
    - how old was the injury when you viewed it and if it took a long time, why?
    - anything else you can tell me about the pathology??
- Treatments
  - What treatments did you use or see used (if you saw, by whom)?
  - What treatments worked and didn’t work (with worked being were able to resolve injuries or lead to retention of livestock that would otherwise be culled or slaughtered)
  - Were there prognostic indicators about survival verse death or euthanasia?
  - What triggered you to euthanase?
  - What triggered salvage slaughter?
  - What proportion of cases were euthanased or went to salvage slaughter compared with were retained by producer?
- What protected livestock from burning
  - In your recollection, were there any differences between farms that burnt with injured livestock compared to farms that burnt without injured livestock?
- *Probe the following factors, whether or not they initially report differences between farms.*
  - - Management factors
    - Previous fire history
    - Landscape (e.g. hilly)
    - Vegetation
    - Luck (e.g. fire reached the farm on a certain day)
    - Fire fighting actions by you or firefighting personnel
    - Other?
- Farm recovery
  - What features of farmer behaviour/management and support do you think were beneficial to a farms recovery after a fire?
    - E.g. feed budgeting, financial grants, consultancy advice, sending stock away etc.
